# Reproductive Outcomes After Breast Cancer in Women With vs Without Fertility Preservation

**DOI:** 10.1001/jamaoncol.2020.5957

**Published:** 2020-11-19

**Authors:** Anna Marklund, Frida E. Lundberg, Sandra Eloranta, Elham Hedayati, Karin Pettersson, Kenny A. Rodriguez-Wallberg

**Affiliations:** 1 Department of Oncology-Pathology, Karolinska Institutet, Stockholm, Sweden; 2 Department of Obstetrics and Gynecology, Södersjukhuset, Stockholm, Sweden; 3 Medical Epidemiology and Biostatistics, Karolinska Institutet, Stockholm, Sweden; 4 Clinical Epidemiology Division, Department of Medicine Solna, Karolinska Institutet, Stockholm, Sweden; 5 Medical Unit of Breast Cancer Sarcoma and Endocrine Tumors, Theme Cancer, Karolinska University Hospital, Stockholm, Sweden; 6 Department of Women’s Health, Karolinska University Hospital, Stockholm, Sweden; 7 Department of Clinical Science, Intervention and Technology, Karolinska Institutet, Stockholm, Sweden; 8 Department of Reproductive Medicine, Division of Gynecology and Reproduction, Karolinska University Hospital, Stockholm, Sweden; 9 Laboratory of Translational Fertility Preservation, BioClinicum J 5:30, Stockholm, Sweden

## Abstract

**Importance:**

The practice of fertility preservation (FP) in women with breast cancer (BC) is spreading, but long-term reproductive outcomes after FP are largely unknown.

**Objective:**

To investigate the long-term reproductive outcomes in women who did or did not undergo FP at the time of BC diagnosis.

**Design, Setting, and Participants:**

A Swedish nationwide cohort study was conducted to investigate the long-term reproductive outcomes of women with BC receiving FP at 1 of the regional FP programs from 1994 to 2017 (n = 425). Population comparators with BC but without history of FP (n = 850) were sampled from regional BC registers, matched on age, calendar period of diagnosis, and county. Data on live births, assisted reproductive technology (ART) use, and mortality were retrieved from population-based registers. Data analysis was performed from January to September 2020.

**Exposures:**

History of having received FP compared with no history of FP (unexposed).

**Main Outcomes and Measures:**

The primary outcome was hazard ratios (HRs) of live births and ART treatments following BC in women with vs without FP and the cumulative incidence of these events in the presence of the competing risk of death.

**Results:**

Women who had undergone FP (n = 425) had lower parity (302 [71.1%] were nulliparous compared with 171 [20.1%] in the unexposed group), were younger (mean [SD] age, 32.1 [4.0] vs 33.3 [3.6] years), more often had estrogen receptor–positive tumors (289 [68.0%] vs 515 [60.6%]), and were more often scheduled for chemotherapy (399 [93.9%] vs 745 [87.7%]). Of 425 women exposed to FP, 97 (22.8%) had at least 1 post-BC live birth (mean follow-up, 4.6 years), compared with 74 of 850 women (8.7%) unexposed to FP (mean follow-up, 4.8 years). Overall, live birth rates after BC were significantly higher among women with FP (adjusted hazard ratio [aHR], 2.3; 95% CI, 1.6-3.3). The 5-year and 10-year cumulative incidence of post-BC live births was 19.4% and 40.7% among FP-exposed women vs 8.6% and 15.8% among comparators, respectively. Rates of ART use were also higher in the FP group (aHR, 4.8; 95% CI, 2.2-10.7). The all-cause mortality rate was lower in women exposed to FP (aHR, 0.4; 95% CI, 0.3-0.7), with 5-year cumulative incidence of death of 5.3% (95% CI, 3.1%-9.0%) vs 11.1% (95% CI, 8.7%-14.1%) for women with vs without FP.

**Conclusions and Relevance:**

In this cohort study of Swedish women after a BC diagnosis, successful pregnancy after BC was possible both in women with and without FP at the time of diagnosis, but a significantly higher likelihood of post-BC live births and ART treatments was observed in women who underwent FP, without any negative association with all-cause survival. This information is valuable for health care clinicians responsible for oncologic treatment and reproductive counseling of women diagnosed with breast cancer at reproductive age.

## Key Points

**Question:**

What are the long-term reproductive outcomes after breast cancer in women with vs without a history of fertility preservation?

**Findings:**

In this population-based nationwide cohort study of 425 Swedish women with breast cancer who underwent fertility preservation, fertility preservation at the time of breast cancer diagnosis was associated with a significantly higher rate of postdiagnosis live births and assisted reproduction treatments, without any negative association with all-cause survival following fertility preservation.

**Meaning:**

The findings of this study may be relevant for reproductive counseling of women with breast cancer diagnosed at reproductive age.

## Teaser

This cohort study investigates the long-term reproductive outcomes in women who did or did not undergo fertility preservation at the time of breast cancer diagnosis.

## Introduction

Breast cancer (BC) is the most common malignant neoplasm diagnosed in women.^1^ Nearly 10% of BC cases occur in women younger than 45 years,^2^ with a 5-year relative survival of approximately 90%.^3^ About half of young women with BC wish to become pregnant after completing therapy.^4,5^ Their chances of subsequent pregnancy are reported to be 40% to 60% lower than in the general population.^6,7^ With improved survival rates, issues of fertility and reproduction among young women with cancer have gained increased attention.^8,9^

Currently, cryopreservation of oocytes and embryos after controlled ovarian stimulation is the standard strategy for fertility preservation (FP) in adult women.^10^ Several recent studies have reported that ovarian stimulation for FP in the setting of BC is safe with regard to relapse-free and overall survival.^11,12,13^

Until now, few studies have evaluated the long-term reproductive outcomes of FP in young women with BC. In a US retrospective study, 10.3% of women with a history of FP for cancer returned to use cryopreserved specimens, and 51.7% of these women had a live birth.^14^ In the Netherlands, a 5-year live birth rate of 27% was reported in a cohort of women with BC, mostly after spontaneous pregnancies.^15^ A Swedish prospective cohort reported a return rate of 21% following FP for BC, with a 26% delivery rate (including spontaneous conceptions) among those who returned, and 5% among those who did not.^16^

In some countries, FP can be costly, and the procedures needed for FP may add additional psychological pressure at the time of cancer treatment planning, thus the need for accurate information on the chances of pregnancy and live birth following BC, both with and without the help of FP. As randomized clinical trials of FP vs no FP in women with cancer facing infertility risk are not feasible, population-based studies that investigate long-term real-world outcomes in unselected patient populations can provide valuable information in this context. The aim of this prospective matched nationwide cohort study was to evaluate the likelihood of live births and performance of assisted reproductive technology (ART) treatments post-BC in women who have vs have not undergone FP.

## Methods

### Data Sources and Study Population

We identified all 468 women with BC who underwent FP (exposed) at 1 of 7 Swedish university hospitals between January 1, 1994, and June 30, 2017. Data on FP procedures were extracted from the electronic medical records of each hospital (eFigure 1 in the Supplement). Details on FP counseling and FP procedures for women with BC in Sweden have been previously reported.^16,17^ Patient consent was obtained at the time of FP, orally until 2008 and thereafter in writing. The Regional Ethics Committee in Stockholm approved the study (Dnr 2011/1758-31/2, amendments 2014/470-32, 2014/1360-32, 2014/1825-32, 2018/275-32 and 2018/1453-32). This study followed the Strengthening the Reporting of Observational Studies in Epidemiology (STROBE) reporting guideline.

Three Swedish quality registers for BC were used to identify the matched cohort. The exposed group was identified in the respective register, and 2 comparators unexposed to FP were sampled for each exposed woman, matched on age group at diagnosis (5-year periods), time of diagnosis (3-year periods), and health care region. For women diagnosed from 2008 to 2017, data were obtained from the Swedish National Quality Register for BC, initiated in 2008.^18^ Data of women diagnosed in 2007 or earlier were obtained from the regional BC registers for the Stockholm-Gotland and West regions. Three women outside these regions were exposed to FP before 2008 and were excluded because we were unable to sample comparators.

**Figure 1.**
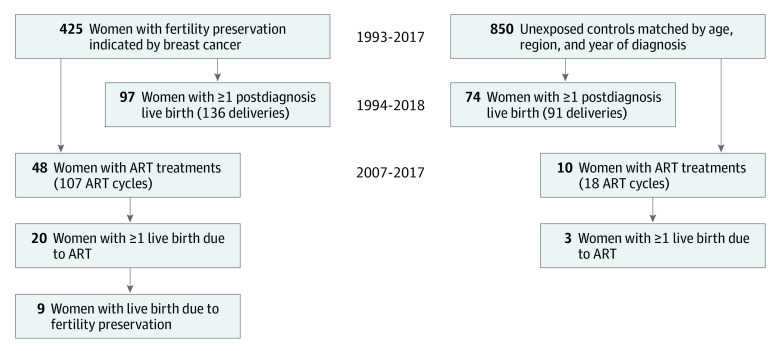
Study Diagram ART indicates assisted reproductive technologies.

Women with cancer in situ, distant metastasis at diagnosis, T4 tumors, synchronic bilateral BC, and without surgery for their BC, and those who could not be identified in any BC register, were excluded, leaving 425 exposed and 850 unexposed women eligible for the study (Figure 1; eFigure 2 in the Supplement).

The cohort was linked to Swedish population registers (eTable 1 in the Supplement) using the personal identity number assigned to all Swedish residents^19^ to retrieve the following data: date of BC diagnosis, age at diagnosis, tumor characteristics and BC treatment details; highest attained educational level and country of birth; treatment details and outcome of all ART cycles since 2007; date of live births before and up to 10 months after diagnosis, date, perinatal and obstetric outcomes of post-BC live births; year of all live births, date of death, and migrations. Year of live births, date of death, and migrations were available until December 31, 2018. All other variables were updated until December 31, 2017.

### Statistical Analysis

Demographic and clinical characteristics of patients were compared using Student *t* tests and Mann-Whitney *U* tests for continuous data and χ^2^ tests for categorical data. All statistical tests were 2-sided with a significance level of .05. Data analysis was performed from January to September 2020.

Hazard ratios (HRs) and 95% CIs for live births, ART use, and all-cause mortality were estimated using left truncated Cox proportional hazard models, with time since diagnosis as the underlying time scale. The date of entry was 10 months after BC diagnosis in analyses of live births and mortality, and 5 months after BC diagnosis in analyses of ART. Person-years at risk were accrued from date of entry until the date of the event of interest or censored at death (for models of birth and ART), emigration, or end of follow-up (December 31, 2017, for ART and December 31, 2018, for live births and all-cause mortality), whichever occurred first. The adjusted models included age at diagnosis, calendar period and parity at diagnosis, country of birth, education level, tumor size, lymph node metastases, estrogen receptor status, and a binary indicator for chemotherapy, categorized according to eTable 2 in the Supplement. The proportional hazards assumption was evaluated using the Schoenfeld residuals from the models.

The cumulative incidence of post-BC childbirths and ART treatments in the presence of the competing risk of death were estimated nonparametrically for exposed and unexposed women. Results are expressed as probabilities of live birth and ART use after BC and presented for all women combined and stratified by parity. The analyses were performed using the statistical software Stata, version 15 (StataCorp).^20^

## Results

The demographic characteristics of the cohort at baseline are presented in eTable 2 in the Supplement. Women who had undergone FP had lower parity (302 [71.1%] were nulliparous compared with 171 [20.1%] in the unexposed group), were younger (mean [SD] age, 32.1 [4.0] vs 33.3 [3.6] years), more often had estrogen receptor–positive tumors (289 [68.0%] vs 515 [60.6%]), and were more often scheduled for chemotherapy (399 [93.9%] vs 745 [87.7%]).

### Childbirth After Treatment of BC

In total, 97 in the FP group (22.8%) (mean follow-up, 4.6 years) and 74 women (8.7%) in the unexposed group (mean follow-up, 4.8 years) had a live birth after BC, when followed-up until December 31, 2018. Compared with women unexposed to FP, those who had undergone FP had more than 2-fold higher rate of live births after diagnosis (HR, 2.6; 95% CI, 1.9-3.5; adjusted HR [aHR], 2.3; 95% CI, 1.6-3.3) (Table).

**Table.** Long-term Reproductive Outcomes and All-Cause Mortality

Outcome	No. of events	Person-years	HR (95% CI)	
			Model 1^a^	Model 2^b^
Post-BC live birth^c^				
Unexposed to FP	74	3753	1 [Reference]	1 [Reference]
Exposed to FP	97	1865	2.6 (1.9-3.5)	2.3 (1.6-3.3)
Post-BC ART treatment^d^				
Unexposed to FP	10	4028	1 [Reference]	1 [Reference]
Exposed to FP	48	2096	9.5 (4.8-18.7)	4.8 (2.2-10.7)
All-cause mortality^c^				
Unexposed to FP	110	4437	1 [Reference]	1 [Reference]
Exposed to FP	27	2477	0.4 (0.3-0.7)	0.4 (0.3-0.7)

Abbreviations: ART, assisted reproductive technology; BC, breast cancer; FP, fertility preservation; HR, hazard ratio.

^a^ Adjusted for time since diagnosis.

^b^ Adjusted for time since diagnosis, age, country of birth, education, parity at diagnosis, calendar period, tumor size, lymph node metastases, estrogen receptor status, and chemotherapy.

^c^ Until December 31, 2018.

^d^ From 2007 to 2017.

**Figure 2.**
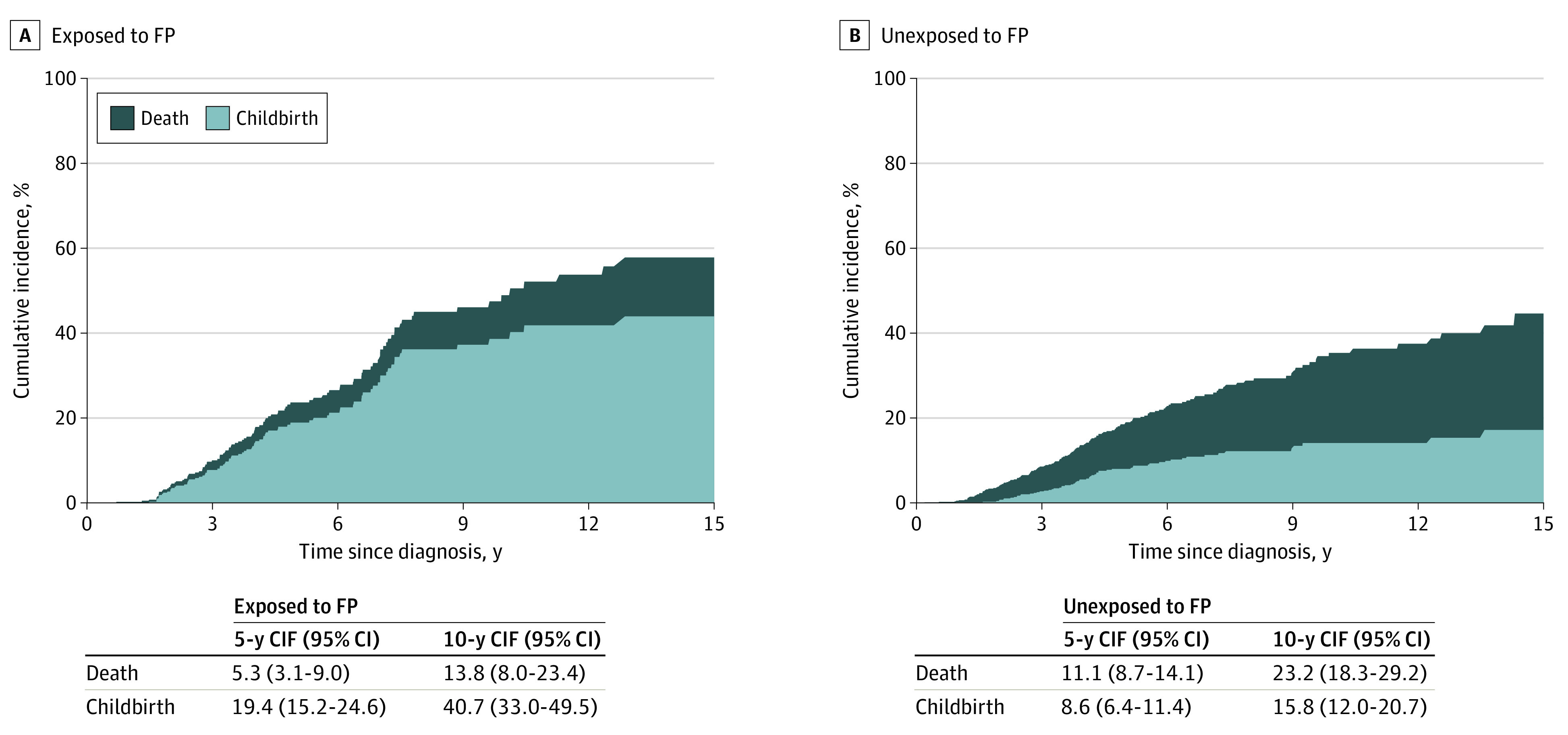
Cumulative Incidence of Childbirth After Breast Cancer by Years Since Diagnosis, With Death as a Competing Risk CIF indicates cumulative incidence function; FP, fertility preservation.

The 5-year cumulative incidence of childbirth was 19.4% (95% CI, 15.2%-24.6%) for exposed women and 8.6% (95% CI, 6.4%-11.4%) for unexposed women, and the corresponding 10-year cumulative incidence was 40.7% (95% CI, 33.0%-49.5%) vs 15.8% (95% CI, 12.0%-20.7%), respectively (Figure 2). Among women who were nulliparous at the time of BC diagnosis, 5-year cumulative incidence of live birth was 19.0% (95% CI, 14.2%-25.1%) in the exposed group and 12.7% (95% CI, 7.6%-21.1%) in the unexposed group, while it was 20.2% (95% CI, 12.7%-31.3%) and 7.5% (95% CI, 5.3%-10.5%), respectively, for women who had given birth to at least 1 child prior to the diagnosis of BC (eFigure 3 in the Supplement). Among women with at least 1 post-BC live birth, 89 (91.8%) received chemotherapy and 41 (42.3%) received adjuvant endocrine therapy in the exposed group compared with 58 (78.4%) and 29 (39.2%), respectively, among unexposed comparators (no statistically significant differences between groups).

### Perinatal Outcomes

Perinatal outcomes were available for all except 1 live birth up to December 31, 2017 (eTable 3 in the Supplement). The HRs for post-BC childbirth were similar when restricting follow-up to the end of 2017 (HR, 2.8; 95% CI, 2.0-3.8; aHR, 2.4; 95% CI, 1.6-3.7). Mean (SD) time from diagnosis to live birth was 4.5 (2.5) years for exposed women and 4.5 (2.3) years for unexposed women. Among the women who gave birth after BC, 77.1% in the exposed group and 33.9% in the unexposed group were nulliparous before their cancer diagnosis. Women with FP were more likely to have more than 1 child after diagnosis (37.3%) compared with women without FP (17.7%). No case of intrauterine fetal death was reported. In the exposed group, 3 women delivered twins at their first post-BC childbirth. Preterm birth (all of them between gestational weeks 32-37) occurred in 3 (3.6%) vs 2 (3.2%) births in the exposed vs the unexposed group.

### ART Treatments After BC

In total, 10 women in the unexposed group (1.2%) and 48 (11.3%) in the exposed group received at least 1 post-BC ART-treatment from 2007 to 2017 (Figure 1). Women with a history of FP had a higher rate of post-BC ART compared with those without FP: HR, 9.5 (95% CI, 4.8-18.7), when adjusted only for time since diagnosis, and aHR, 4.8 (95% CI, 2.2-10.7), after additional adjustment (Table). The mean (SD) follow-up time was 4.9 (4.1) vs 4.7 (4.0) years in the exposed vs unexposed groups.

Characteristics and reproductive outcomes of women with at least 1 post-BC ART treatment are presented in eTable 4 in the Supplement. The live birth rate with ART was 30% (3 of 10) in women without FP and 42% (20 of 48) in women with FP (difference not significant), where 43% (9 of 21) of children were conceived using oocytes/embryos obtained from FP treatments. In the exposed group, 58% (62 of 107) of all post-BC ART treatments consisted of transfer of frozen-thawed embryos compared with 22% (4 of 18) in the unexposed group. Treatment with donor oocytes was given to 1 woman in FP group, without resulting in a pregnancy.

### All-Cause Mortality

In this cohort, 27 women (6.4%) in the exposed group and 110 women (12.9%) in the unexposed group had died by the end of follow-up. The mean (SD) follow-up time was 5.8 (4.2) vs 5.2 (4.0) years in the exposed vs unexposed group. The rate of all-cause mortality was lower in the exposed group: aHR, 0.4 (95% CI, 0.3-0.7) (Table). The 5-year cumulative incidence of death was 5.3% (95% CI, 3.1%-9.0%) in the exposed group and 11.1% (95% CI, 8.7%-14.1%) in the unexposed group, and the corresponding 10-year cumulative incidence was 13.8% (95% CI, 8.0%-23.4%) vs 23.2% (95% CI, 18.3%-29.2%).

## Discussion

This nationwide study found generally reassuring post-BC reproductive outcomes among young female patients with BC. Fertility preservation was associated with significantly higher rate of post-BC live births and use of ART treatments without an association with lower all-cause survival.

In the available literature, the pregnancy rate in women with a history of BC has been reported to be, on average, 40% to 60% lower than in general population.^7,21^ Data on long-term reproductive outcomes after cancer treatment in women who received FP are currently scarce. In what is to our knowledge the largest reported cohort of women counseled on FP at the time of BC diagnosis (n = 118), a 5-year live birth rate of 29.4% vs 19% was reported among those who proceeded to vs those who declined FP.^15^ In our study, the cumulative incidence of post-BC live births 5 years after diagnosis was 19% vs 9% among women with vs without history of FP, and after 10 years it was 41% and 16%, respectively. For the first post-BC live birth, at least 20% of the pregnancies in the exposed group and 4% in the unexposed group were achieved through ART. These results indicate generally reassuring long-term reproductive outcomes in women diagnosed with BC during their reproductive years but also highlight the importance of FP counseling in this population. Moreover, previous studies reporting live birth rates in women with a history of cancer often include the births achieved with the help of gestational carriers.^14,22^ This procedure is not permitted in Sweden; therefore, in countries that allow surrogacy, the rate of post-BC live births among women with BC may be higher than in the present study.

### Limitations and Strengths

Lack of data on childbearing intent or wish at the time of BC diagnosis is an important limitation of this study, as women who wish to have children after completion of BC treatment are more prone to opt for FP, potentially leading to confounding by indication. Younger age and lower parity at diagnosis, as well as higher rate of post-BC ART treatments in women who have undergone FP, also support that assumption. Additional adjustment of our results for childbearing intent would probably rend the difference in birth rate between the 2 groups smaller, as suggested by a previous intervention study of post-BC births.^23^ Another limitation is that we had access to data on pregnancies that resulted in live births and on use of ART but no data on natural conceptions that did not result in live births. Miscarriages and abortions are not systematically registered in the Swedish registers, and many women who experience early miscarriage never contact a caregiver. Therefore, we are not able to elaborate on the natural post-BC conception rate.

Women exposed to FP had a lower cumulative incidence of death following BC diagnosis in our study; however, disease-specific mortality and disease-free survival could not be investigated. Previous studies have indicated at least noninferior disease-free and overall survival in women with BC who undergo FP, adding to the evidence of safety of the FP.^11,12,14^ Still, when discussing better prognosis among women who become pregnant after BC treatments,^24,25^ the selection bias known as “healthy mother effect” is often mentioned.^26^ Possibly, a similar “healthy FP effect” would be plausible, as the women who prefer to undergo FP treatments could appreciate their disease as transitory with good chances of survival, whereas other women, more affected by the disease, could have chosen to skip additional medical procedures. We have thus adjusted our analysis for disease-related variables, but there still could be other prognostic factors that we could not capture. Whether the proposed healthy FP effect indeed exists, and whether FP is associated with disease-free survival, should be further investigated in large studies of FP safety in the setting of BC in young women.

Strengths of the current study include a large nationwide sample with long follow-up and the use of prospectively collected data from population-based registers, which also allowed us to obtain a well-characterized group of comparator women with BC unexposed to FP. In general, the current scientific literature lacks studies with appropriate control groups for the evaluation of long-term reproductive outcomes in women with a history of FP indicated by cancer treatments. Moreover, many retrospective review studies present self-reported data on pregnancies and births, whereas the register-based data extracted for this study are more robust. An additional strength is that cancer care and FP indicated by medical reasons are provided within the public tax-funded system in Sweden, ensuring equal access to all citizens and reducing the risk of selection bias in the exposed group.

## Conclusions

There is an obvious clinical value in describing reproductive patterns in survivors of BC with and without history of FP. In the settings of FP services for women with BC, decisions should be based on accurate information regarding the chances of biological parenthood following BC, both with and without the help of FP. Results of this nationwide cohort study indicate that although successful pregnancy after BC is possible both in women with and without FP, FP is associated with significantly higher rates of post-BC live births and use of ART treatments, without any deleterious association with all-cause survival during a mean follow-up of 5.2 years. These findings add to the current knowledge regarding FP treatments in women with BC.

## Notes

Supplement. **eTable 1.** Swedish population registers used to obtain information on cohort. **eTable 2.** Characteristics of women with breast cancer (BC) in the matched cohort. **eTable 3.** Characteristics of women with ≥1 live birth > 10 months after diagnosis of breast cancer, and perinatal outcome for the first live birth if >1 (data for 1995-2017). **eTable 4.** Characteristics and reproductive outcome of women with ART-treatments after diagnosis of BC (data for treatments available for years 2007-2017). **eFigure 1.** Choice of fertility preservation method among women with breast cancer. **eFigure 2.** Study diagram. **eFigure 3.** Cumulative incidence of childbirth after breast cancer by years since diagnosis, stratified on parity at diagnosis, with death as a competing risk.
