# Supplementary material for: Reproductive Outcomes After Breast Cancer in Women With vs Without Fertility Preservation
Source: JAMA Oncol. 2020 Nov 19;7(1):1–6. doi: 10.1001/jamaoncol.2020.5957 (PMC7677871; doi:10.1001/jamaoncol.2020.5957)
Supplement: Supplement. — eTable 1. Swedish population registers used to obtain information on cohort. eTable 2. Characteristics of women with breast cancer (BC) in the matched cohort. eTable 3. Characteristics of women with ≥1 live birth > 10 months after diagnosis of breast cancer, and perinatal outcome for the first live birth if >1 (data for 1995-2017). eTable 4. Characteristics and reproductive outcome of women with ART-treatments after diagnosis of BC (data for treatments available for years 2007-2017). eFigure 1. Choice of fertility preservation method among women with breast cancer. eFigure 2. Study diagram. eFigure 3. Cumulative incidence of childbirth after breast cancer by years since diagnosis, stratified on parity at diagnosis, with death as a competing risk. [file jamaoncol-e205957-s001.pdf]

Supplemental Online Content

Marklund A, Lundberg FE, Eloranta S, Hedayati E, Pettersson K, Rodriguez-Wallberg KA. Reproductive outcomes after breast cancer in women with vs without fertility preservation. *JAMA Oncol*. Published November 19, 2020.
doi:10.1001/jamaoncol.2020.5957

eTable 1. Swedish population registers used to obtain information on cohort.
eTable 2. Characteristics of women with breast cancer (BC) in the matched cohort.
eTable 3. Characteristics of women with ≥1 live birth > 10 months after diagnosis of breast cancer, and perinatal outcome for the first live birth if >1 (data for 1995-2017).
eTable 4. Characteristics and reproductive outcome of women with ART-treatments after diagnosis of BC (data for treatments available for years 2007-2017).
eFigure 1. Choice of fertility preservation method among women with breast cancer.
eFigure 2. Study diagram.
eFigure 3. Cumulative incidence of childbirth after breast cancer by years since diagnosis, stratified on parity at diagnosis, with death as a competing risk.

This supplemental material has been provided by the authors to give readers additional information about their work.

© 2020 Marklund et al. *JAMA Oncology.*

eTable 1. Swedish population registers used to obtain information on cohort.

Register	Description	Years	Information retrieved
Swedish National Cancer Register	Since 1958, six regional cancer centers report on all cancer cases, and the data includes histological type, site and stage of tumor, date of diagnosis, eventual date and cause of death, with the coverage rate of 96%.[1]	1994-2017	date of BC diagnosis, age at diagnosis, tumor characteristics, treatment details for BC.
Swedish National Quality Register for BC	A population-based register, with information on tumor characteristics, treatment, and relapse occurrence in patients diagnosed with invasive BC since 2008, with the coverage rate of 99%.[2]	2008-2017	
Regional quality registers for BC, for regions West and Stockholm-Gotland	Prior to 2008, data on tumor characteristics, treatment details and relapse occurrence for all cases of invasive BC were reported to the regional quality registers.	1994-2007	
The Total Population Register	It contains data on life events including birth, death, marital status and migration; maintained by the government agency Statistics Sweden. Updates are transmitted daily from the Tax Agency to the TPR.3	1994-2018	date of death, immigrations and emigrations
The Swedish Multi-Generation Register	It contains connections between index persons (registered in Sweden at some time since 1961 and born in 1932 or later) and their biological parents.[4]	1994-2018	year of livebirths
Longitudinal integrated database for health insurance and labor market studies (LISA)	It is maintained by the government agency Statistics Sweden and holds annual registers since 1990, integrating existing data from the labor market, educational and social sector.[5]	1994-2017	educational level and country of birth
The Medical Birth Register	It includes data on all births in Sweden since 1973, reported information comes from medical records from the prenatal, delivery and neonatal care.[6]	1994-2017	date, perinatal and obstetric outcomes of post-diagnosis livebirths
The National Quality Registry for Assisted Reproduction	It was started 2007 and contains data on all ART-treatments in Sweden, in both public and private IVF clinics, with 100% coverage rate of care providers.[7]	2007-2017	treatment details and outcome of all ART cycles
Abbreviations: ART, assisted reproductive technology; BC, breast cancer.			

References:
1. Socialstyrelsen. Swedish National Cancer Register. Available from: https://www.socialstyrelsen.se/en/statistics-and-data/registers/register-information/swedish-cancer-register/. [Accessed 1st June 2020]
2. Lofgren L, Eloranta S, Krawiec K, Asterkvist A, Lonnqvist C, Sandelin K, et al. Validation of data quality in the Swedish National Register for Breast Cancer. BMC Public Health. 2019;19(1):495.
3. Ludvigsson JF, Almqvist C, Bonamy AK, Ljung R, Michaelsson K, Neovius M, et al. Registers of the Swedish total population and their use in medical research. Eur J Epidemiol. 2016;31(2):125-36.
4. Ekbom A. The Swedish Multi-generation Register. Methods Mol Biol. 2011;675:215-20.
5. Ludvigsson JF, Svedberg P, Olen O, Bruze G, Neovius M. The longitudinal integrated database for health insurance and labour market studies (LISA) and its use in medical research. Eur J Epidemiol. 2019;34(4):423-37.
6. Axelsson O. The Swedish medical birth register. Acta Obstet Gynecol Scand. 2003;82(6):491-2.
7. The National Quality Registry for Assisted Reproduction. Available from: https://www.medscinet.com/qivf/. [Accessed 1st June 2020]

© 2020 Marklund et al. *JAMA Oncology.*

eTable 2. Characteristics of women with breast cancer (BC) in the matched cohort.

Characteristic	Exposed to FP (n=425)	Unexposed to FP (n=850)	*P*
Educational level			
Compulsory school	27 (6.4%)	84 (9.9%)	
Secondary school	151 (35.5%)	290 (34.1%)	0.217
Higher education	244 (57.4%)	470 (55.3%)	
Missing	3 (0.7%)	6 (0.7%)	
Country of birth			
Nordic	345 (81.2%)	628 (73.9%)	0.004
Non-Nordic	80 (18.8)	222 (26.1%)	
Age at diagnosis, years			
21-29	115 (27.1%)	128 (15.1%)	<0.001
30-34	179 (42.1%)	379 (44.6%)	
35-42	131 (30.8%)	343 (40.3%)	
Geographical region			
Stockholm Gotland	226 (53.2%)	452 (53.2%)	
West region	64 (15.1%)	128 (15.1%)	1.0
Other	135 (31.7%)	270 (31.7%)	
Parity at BC diagnosis			<0.001
0	302 (71.1%)	171 (20.1%)	
1	102 (24%)	183 (21.5%)	
>=2	21 (4.9%)	496 (58.5%)	
Year of diagnosis			0.100
1994-2007	72 (16.9%)	144 (16.9%)	
2008-2017	352 (83.1%)	706 (83.1%)	
Tumor size			0.036
T0	15 (3.5%)	27 (3.2%)	
T1	184 (43.3%)	352 (41.4%)	
T2	190 (44.7%)	341 (40.1%)	
T3	34 (8.0%)	119 (14.0%)	
TX (size cannot be assessed)	2 (0.5%)	11 (1.3%)	
Lymph nodes with metastasis			0.035
0	271(63.8%)	478 (56.2%)	
1-3	120 (28.2%)	271 (31.9%)	
>3	34 (8.0%)	99 (11.7%)	
Missing	0 (0%)	2 (0.2%)	
Tumor Grade			0.178
1	26 (6.1%)	51(6.0%)	
2	108 (25.4%)	177 (20.8%)	
3	180 (42.4%)	359 (42.2%)	
Missing	111 (26.1%)	263 (30.9%)	
ER-status			0.034
Positive	289 (68.0%)	515 (60.6%)	
Negative	128 (30.1%)	313 (36.8%)	
Missing	8 (1.9%)	22 (2.6%)	
PR-status			0.029
Positive	249 (58.6%)	431 (50.7%)	
Negative	167 (39.3%)	397 (46.7%)	
Missing	9 (2.1%)	22 (2.6%)	
HER-2			
Amplified	108 (25.4%)	176 (20.7%)	
Non-amplified	199 (46.8%)	429 (50.5%)	0.158
Unknown	118 (27.8%)	245 (28.8%)	

© 2020 Marklund et al. *JAMA Oncology.*

Characteristic	Exposed to FP (n=425)	Unexposed to FP (n=850)	*P*
**Therapy**			
Neoadjuvant	105 (24.7%)	249 (29.3)	0.100
Adjuvant	419 (98.6%)	823 (96.8%)	0.161
Chemotherapy			0.002
Yes	399 (93.9%)	745 (87.7%)	
No	25 (5.9%)	98 (11.5%)	
Missing	1 (0.2%)	7 (0.8%)	
Radiotherapy			
Yes	317 (74.6%)	650 (76.5%)	
No	94 (22.1%)	141 (16.6%)	0.003
Missing	14 (3.3%)	59 (6.9%)	
Endocrine therapy			0.011
Yes	281 (66.1%)	498 (58.6%)	
No	121 (28.5%)	314 (36.9%)	
Missing	23 (5.4%)	38 (4.5%)	
Her2- therapy			0.040
Yes	111 (26.1%)	169 (19.9%)	
No	253 (59.5%)	549 (64.6%)	
Missing	61 (14.4%)	132 (15.5%)	
NOTE: Data are presented as No (%) unless noted otherwise.<br>Abbreviations: ER, estrogen receptor; FP, fertility preservation; PR, progesterone receptor.			

© 2020 Marklund et al. *JAMA Oncology.*

eTable 3. Characteristics of women with ≥1 live birth > 10 months after diagnosis of breast cancer, and perinatal outcome for the first live birth if >1 (data for 1995-2017).

Characteristic or outcome	Women with FP (n=83, livebirths n= 119)	Women without FP (n=62, livebirths n=75)	*P*
Age at time of delivery, years			
Mean (SD)	35.3 (3.9)	35.2 (3.5)	0.538
Smoking	7 (8.4%)	3 (4.8%)	0.398
BMI			
Mean (SD)	23.8 (3.9)	25.1 (4.0)	0.014
Missing	8 (9)	3 (5)	
Family situation			
Living with a partner	76 (91.6%)	58 (93.5%)	0.318
Time between cancer diagnosis and			
first post-diagnosis livebirth	2 (2.4%)	1 (1.6%)	
< 2 years	50 (60.2%)	36 (58.1%)	0.897
2-5 years	31 (37.4%)	25 (40.2%)	
>5 years			
Parity at 1st post-diagnosis live birth			
0	64 (77.1%)	21 (33.9%)	
1	16 (19.3%)	18 (29.0%)	<0.001
≥2	3 (3.6%)	23 (37.1%)	
Number of post-diagnosis births			
1	52 (62.7%)	51 (82.3%)	
2	26 (31.3%)	9 (14.5%)	0.036
≥3	5 (6%)	2 (3.2%)	
Mode of conception			
ART-treatment	20	1	<0.001
Delivery mode			
Vaginal	44 (53%)	46 (74.2%)	
Assisted vaginal	11 (13.25%)	2 (3.2%)	
Planned cesarean	11 (13.25%)	5 (8.1%)	0.044
Unplanned cesarean	17 (20.5%)	9 (14.5%)	
Gestational age at birth, weeks			
Mean (SD), range	39.6 (1.7), 32-43	39.7 (1.6), 34-42	0.269
Gestational weight at birth, grams			
Mean (SD)	3522 (578)	3568 (487)	0.614
Range	1558-4915	2500-4770	
SGA	2 (2.4%)	0	0.145
LGA	2 (2.4%)	0	0.145
Hypertensive disorders of pregnancy			
Gestational hypertension	3 (3.6%)	1/62 (1.6%)	0.467
Preeclampsia	2 (2.4%)	0/62	0.218
GDM gestational diabetes	1 (1.2%)	0	0.386
Induced delivery	20 (24.1%)	8 (12.9%)	0.091
Apgar <7 at 5 minutes	2 (2.4%)	0	0.218
Congenital malformation	3 (3.6%)	1 (1.6%)	0.467

NOTE: Data are presented as No (%) unless noted otherwise.
Abbreviations: FP, fertility preservation; SD, standard deviation.

© 2020 Marklund et al. *JAMA Oncology.*

eTable 4. Characteristics and reproductive outcome of women with ART-treatments after diagnosis of BC (data for treatments available for years 2007-2017).

**Characteristic or outcome**	**Women with FP (n= 48, ART-treatments, n= 107)**	**Women without FP (n= 10, ART-treatments, n= 18)**	***P***
Time between cancer diagnosis and first post-diagnosis ART-treatment			
<2 years	10 (20.8%)	2 (20%)	
2-5 years	23 (47.9%)	4 (40%)	0.857
>5 years	15 (31.3%)	4 (40%)	
Type of ART-treatment			
IVF (for fresh cycles)	10	3	0.347
ICSI (for fresh cycles)	30	8	0.161
Interrupted	17	5	0.220
FET	62	4	0.005
Use of frozen eggs	20 cycles/15 women	1 cycle	0.080
Donated oocytes	1	0	0.680
Number of ART-treatments/cycles per woman			
1	21 (43.7%)	6 (60%)	
2	10 (20.8%)	2 (20%)	0.36
≥3	17 (35.5%)	2 (20%)	
Outcome per cycle			
ET	90	13	0.220
Biochemical pregnancies	4 (2 women, 4 cycles)	0	0.404
Miscarriage	3 (2 women)	0	0.472
Livebirths	21 (20 women)	3 (3 women)	0.768

NOTE: Data are presented as No (%) unless noted otherwise.
Abbreviations: ART, assisted reproductive technologies; ET, embryo transfer; FET, frozen embryo transfer; FP, fertility preservation; IVF, in vitro fertilization; ICSI intraplasmatic sperm injection.

© 2020 Marklund et al. *JAMA Oncology.*

eFigure 1. Choice of fertility preservation method among women with breast cancer. COS, controlled ovarian stimulation.

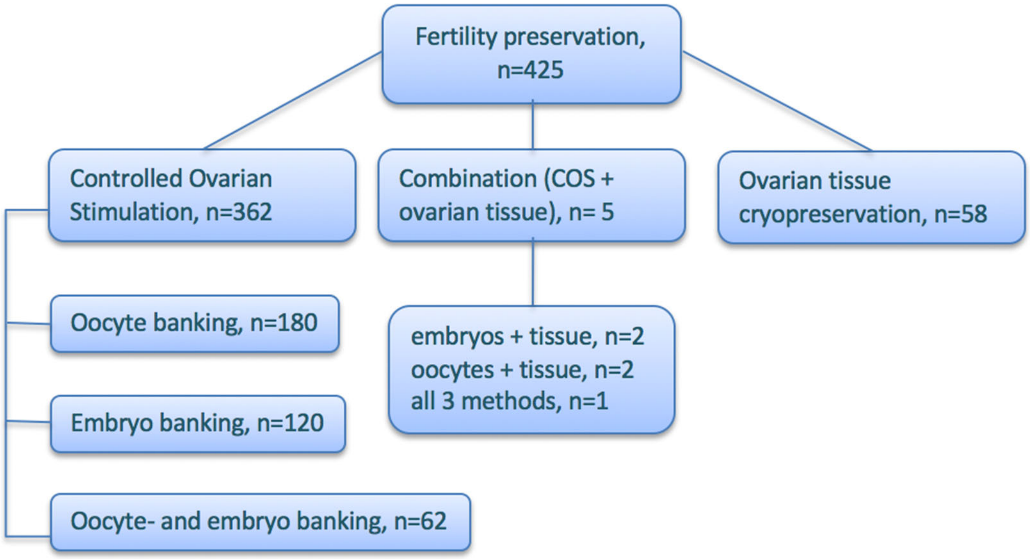

© 2020 Marklund et al. *JAMA Oncology.*

eFigure 2. Study diagram. FP, fertility preservation.

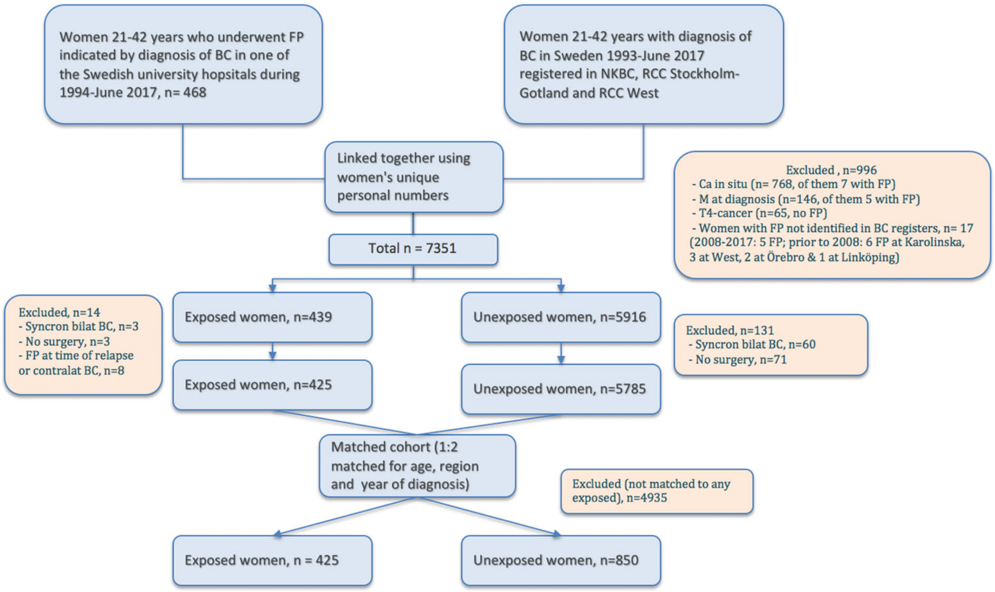

© 2020 Marklund et al. *JAMA Oncology.*

eFigure 3. Cumulative incidence of childbirth after breast cancer by years since diagnosis, stratified on parity at diagnosis, with death as a competing risk. FP, fertility preservation.

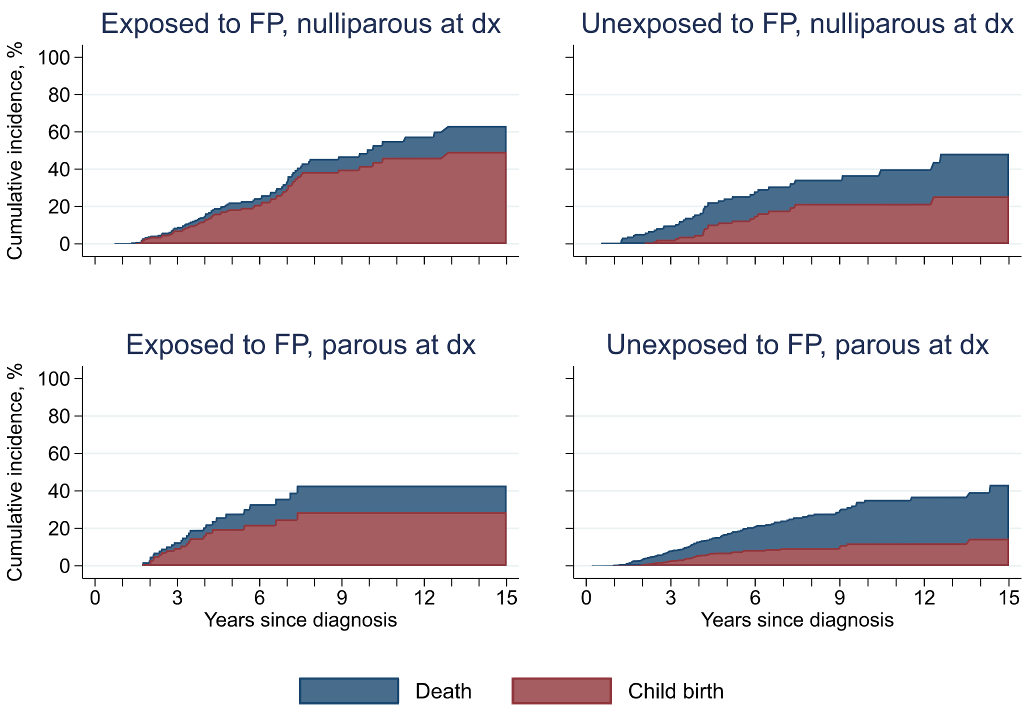

	Exposed to FP		Unexposed to FP	
	5-year CIF (95% CI)	10-year CIF (95% CI)	5-year CIF (95% CI)	10-year CIF (95% CI)
**Nulliparous at diagnosis**				
Death	3.9 (1.9-7.9)	12.9 (6.2-25.8)	13.4 (8.4-20.8)	16.6 (9.8-27.2)
Child birth	19.0 (14.2-25.1)	43.4 (34.3-53.8)	12.7 (7.6-21.1)	24.3 (15.9-36.0)
**Parous at diagnosis**				
Death	9.7 (4.2-21.6)	17.4 (8.2-34.7)	10.5 (7.9-13.9)	25.2 (19.3-32.5)
Child birth	20.2 (12.7-31.3)	30.8 (19.2-47.2)	7.5 (5.3-10.5)	13.4 (9.3-19.0)

© 2020 Marklund et al. *JAMA Oncology.*
